# Supplementary material for: Effects of Vitamin E on Redox balance in regulating thiol/disulfide homeostasis in sepsis: An antioxidant therapy perspective
Source: PLoS One. 2025 Nov 6;20(11):e0336334. doi: 10.1371/journal.pone.0336334 (PMC12591419; doi:10.1371/journal.pone.0336334)
Supplement: Attachment — Submitted filename: Response to Reviewers.docx [file pone.0336334.s003.docx]

**Manuscript ID:** PONE-D-25-12349
**Title:** ***Effects of Vitamin E on Redox Balance in Regulating Thiol/Disulfide Homeostasis in Sepsis: An Antioxidant Therapy Perspective***

Dear Editor and Reviewers,

We would like to sincerely thank you for your careful evaluation of our manuscript and for the insightful and constructive comments. We have revised the manuscript accordingly, and we believe these changes have substantially improved the quality, clarity, and scientific rigor of our work. Below, we provide a detailed, point-by-point response to each comment. All revisions have been incorporated into the updated manuscript, with page numbers indicated.

**Overall Statement:**
We sincerely thank the reviewers for their constructive and insightful feedback. We have carefully revised the manuscript according to the suggestions provided. Below, we present our detailed responses under the specific headings mentioned.

**Reviewer’s Comment:** Clarify the aim of the study and its contribution at the beginning of the Introduction. Explain how vitamin E contributes to redox balance and sepsis pathophysiology compared to previous studies.
**Response:** We thank the reviewer for this valuable suggestion. In the revised Introduction (page 6), we added a clear statement of the study aim and novelty in one concise sentence. We also emphasized the mechanistic role of vitamin E in modulating redox homeostasis and sepsis-induced oxidative stress, highlighting how our work differs from previous studies.

**Reviewer’s Comment:** Justify the rationale for vitamin E administration, experimental conditions, and dose selection. Discuss sample size determination.
**Response:** We have expanded the Methods section (page 8) to provide detailed justification for vitamin E administration, citing previous studies that supported our chosen dose and timing. Sample size was calculated using power analysis (G*Power 3.1), ensuring adequate statistical power to detect significant differences (page 7). This information has now been explicitly added.

**Reviewer’s Comment:** Strengthen rationale for NLR, PLR, CRP, and add more inflammatory/anti-inflammatory markers if possible.
**Response:** In the Results and Discussion sections (page 5-19), we clarified why NLR, PLR, and CRP were chosen as cost-effective and widely validated indicators of systemic inflammation. Additionally, we measured cytokines including TNF-α and IL-40, which provide novel insights into anti-inflammatory effects of vitamin E in sepsis. These findings have been emphasized in the revised Discussion.

**Reviewer’s Comment:** Provide quantitative or semi-quantitative scoring instead of relying solely on descriptive statements.
**Response:** We fully agree. Histopathological findings were rescored using a semi-quantitative scale (0–3) assessing inflammatory infiltration, vascular congestion, and tissue degeneration. These scores are presented in Table 4 and referenced in the Results (page 17), providing robust and reproducible evaluation of tissue injury.

**Reviewer’s Comment:** Ensure consistency in statistical terminology, clarity on correlation analyses, and avoid overinterpretation of non-significant findings.
**Response:** We revised all statistical descriptions for clarity and consistency (page 10). Pearson correlation analyses are reported consistently, and Spearman tests were used for non-parametric distributions. We carefully avoided interpretation of non-significant results. Furthermore, figure legends and tables were updated for accuracy and transparency.

**Reviewer’s Comment:** Improve language clarity, conciseness, and flow.
**Response:** The manuscript was thoroughly revised for English language and style by a professional language editor. We ensured conciseness, precision, and academic tone throughout the text.

**Reviewer’s Comment:** Update references to include the most recent and relevant literature.
**Response:** We updated the reference list to include recent high-quality studies published between 2020–2024 related to oxidative stress, thiol-disulfide homeostasis, and sepsis pathophysiology. Older references were retained only when seminal.

**Detailed responses to reviewers 1, 2 and 3**

**Reviewer 1:**

**Comment :** *The study does not provide sufficient novelty. Many studies on thiol/disulfide homeostasis in humans already exist.*
**Response:** We appreciate this observation. While several human studies have investigated thiol/disulfide homeosta

1. We used an **experimental LPS-induced sepsis model** to assess the protective role of vitamin E, which to our knowledge has not been previously reported.
2. We integrated thiol/disulfide dynamics with **emerging inflammatory markers (IL-40)** alongside conventional markers (CRP, TNF-α).

This dual focus on both classical and emerging biomarkers under controlled experimental conditions highlights a unique mechanistic insight into the antioxidant potential of vitamin E. We have clarified this point in the Introduction (page 4-5) and Discussion (page 19).

**Reviewer 2:**

**Comment 1:** *Keywords are not consistent with MeSH terms.*
**Response:** Corrected. Keywords have been revised to align with MeSH terminology, including “Sepsis,” “Thiol-Disulfide Exchange,” “Vitamin E,” “Inflammation,” and “Oxidative Stress.” (p. 2).

**Comment 2:** *The Introduction is too long and lacks a concise aim.*
**Response:** We shortened the Introduction and added a clear statement of aim at the end:
*“This study aimed to investigate whether vitamin E supplementation could restore thiol–disulfide homeostasis and modulate inflammatory and hematological alterations in an experimental sepsis model.”* (p. 6).

**Comment 3:** *Sample size justification is missing.*
**Response:** We added an explanation:
*“The sample size was based on prior experimental sepsis studies reporting significant differences in redox biomarkers with similar group sizes, as no formal power analysis could be performed in this exploratory design.”* (p. 7).

**Comment 4: *Methods require more clarity.*
Response:** We clarified group allocation, dosing, randomization, the Erel–Neselioglu method for thiol-disulfide measurement, and the histopathological scoring system (p. 6–8).)

**Comment 5:** *Statistical reporting (“Pearson correlation”) contains errors.*
**Response:** Corrected to *“Pearson’s correlation coefficient”*. (p. 10).

**Comment 6:** *NLR and PLR may not be sufficient inflammatory indices.*
**Response:** We acknowledge this limitation and cited recent studies (2020–2024) supporting NLR and PLR as validated indices in sepsis. We have also emphasized the complementary role of IL-40 and TNF-α as novel markers. (p. 13).

**Comment 7:** *Reference list outdated and writing style issues.*
**Response:** References have been updated with recent literature (2019–2024). We also corrected typographical issues and eliminated first-person pronouns to improve academic style.

**Reviewer 3:**

**Comment 1:** *Single dose of vitamin E is a limitation.*
**Response:** We agree. We explicitly mention this in the Discussion:
*“A limitation of this study is the use of a single dose of vitamin E, which does not allow evaluation of dose-dependent effects.”* (p. 20).

**Comment 2:** *No cytokine panel measurement (e.g., IL-6).*
**Response:** TNF-α and IL-40 were measured, but we acknowledge that other cytokines (e.g., IL-6, IL-10) were not evaluated. This limitation has been added to the Discussion (p. 16).

**Comment 3:** *Histopathology is descriptive, not quantitative.*
**Response:** We addressed this by providing semi-quantitative scoring of lung, liver, and kidney pathology. These data are now included as **Table 4** and illustrated in **Figure 1** (p. 17).

**Comment 4:** *Statistical interpretation inconsistent; some non-significant results overstated.*
**Response:** We carefully revised the Results to ensure that only statistically significant differences (p<0.05) are highlighted. Non-significant findings are now clearly stated. (p. 11-18).

**Comment 5:** *High dose clinical translation is questionable.*
**Response:** We added a sentence to the Discussion:
*“While the administered dose demonstrated efficacy in our murine model, extrapolation to clinical settings requires caution, as human therapeutic ranges may differ significantly.”* (p. 20).

**Comment 6:** *Language fluency needs improvement.*
**Response:** The manuscript has undergone extensive language editing by a native-level scientific editor to improve clarity and readability.

We hope these revisions satisfactorily address the reviewers’ concerns. We are confident that the revised manuscript has been substantially improved and now provides a clearer, more rigorous contribution to the field.

Sincerely,

**Assis. Prof. Dr. Veli Fahri Pehlivan**

(on behalf of all authors)
Harran University, Faculty of Medicine
Department of Anesthesia and Reanimation
Osmanbey Campus, PC 63300, Sanlıurfa, Türkiye
Tel: +90 532 7696566
E-mail: vfpehlivan@harran.edu.tr
