# Supplementary material for: Effects of Vitamin E on Redox balance in regulating thiol/disulfide homeostasis in sepsis: An antioxidant therapy perspective
Source: PLoS One. 2025 Nov 6;20(11):e0336334. doi: 10.1371/journal.pone.0336334 (PMC12591419; doi:10.1371/journal.pone.0336334)
Supplement: Attachment — Submitted filename: Response to Reviewers Letters.docx [file pone.0336334.s004.docx]

**Manuscript ID:** PONE-D-25-12349
**Title:** ***Effects of Vitamin E on Redox Balance in Regulating Thiol/Disulfide Homeostasis in Sepsis: An Antioxidant Therapy Perspective***

Dear Editor and Reviewers,

We would like to express our sincere gratitude to the Academic Editor and the reviewers for their careful evaluation of our manuscript and for the constructive comments provided. The feedback we received was highly valuable in improving the scientific rigor, clarity, and overall quality of the study. We carefully considered each point raised and revised the manuscript accordingly. We believe that the improvements made in response to these suggestions have substantially strengthened the manuscript and enhanced its contribution to the field. We truly appreciate the time and expertise invested in the review process, and we are confident that the revised version now addresses the concerns raised.

**Reviewer 2:**

**We sincerely thank Reviewer 2 for the careful and constructive evaluation of our manuscript. Your insightful comments and suggestions were invaluable in guiding the revision process. Below, we provide point-by-point responses to each of your remarks, keeping the original comments unchanged for clarity. We have addressed all points raised to the best of our ability, and the corresponding revisions have been incorporated into the manuscript. We believe that these changes have improved the clarity, rigor, and overall scientific contribution of our study.**

**Comment 1: *“Please ensure the existence of each keyword in MeSH browser.”***

**Response 1 :** We carefully checked each keyword against the MeSH browser. Non-standard terms were revised, and all selected keywords now correspond to valid MeSH entries. The updated keywords are included in the revised manuscript.

**Comment 2: *“The introduction could be more concise.”***

**Response 2:** The Introduction has been streamlined by removing redundancies and shortening overly descriptive sentences. This has improved conciseness while maintaining context for the research question.

**Comment 3: *“Please provide one clear aim sentence.”***

**Response** **3:** We added a single, clear aim statement at the end of the Introduction:
**“This study aimed to investigate the protective effects of vitamin E on oxidative stress, thiol–disulfide homeostasis, hematological indices, cytokine responses, and histopathological alterations in an experimental sepsis model.”**

**Comment 4. *“Please add the novelty of the study.”***

**Response** **4:** We highlighted the novelty in the Introduction and Discussion: this is the **first experimental study to demonstrate the modulatory effects of vitamin E on IL-40, a newly identified cytokine**, in addition to its established antioxidant role. This unique finding positions IL-40 as a potential therapeutic biomarker in sepsis.

**Comment 5. *“Methods: Please provide the design of the study clearly.”***

**Response** **5:** The Methods section has been revised to explicitly state that this is a **randomized, controlled experimental animal study**, with four groups (Control, Sepsis, Vitamin E, Sepsis + Vitamin E). Randomization and blinding procedures have been described more clearly.

**Comment 6. *“How was the sample size arrived at?”***

**Response 6:** We added a clarification to the Methods: the sample size (n=8 per group) was determined based on similar prior experimental studies investigating antioxidant interventions in murine sepsis, while also considering ethical limitations on animal use.

**Comment 7. *“‘For correlation, the pearson correlation test was used’ → Pearson's, please.”***

**Response** **7**: This has been corrected to **“Pearson’s correlation test”** throughout the manuscript.

**Comment 8. *“NLR, PLR are used in the study. Are these enough to reflect the inflammatory status? Please, to back it up, add up to date references on their role in inflammatory conditions such as ankylosing spondylitis.”***

**Response** **8**: We agree and have expanded the Discussion with updated references supporting the role of NLR and PLR as systemic inflammatory markers in sepsis and other inflammatory diseases, including ankylosing spondylitis (e.g., PMID: 36986479, PMID: 32000859). This provides stronger justification for their inclusion.

**Comment 9. *“Please update and diversify the reference list.”***

**Response 9:** We reviewed and updated the reference list, adding recent publications (2020–2024) relevant to thiol–disulfide homeostasis, cytokines, and inflammatory indices in sepsis and other systemic conditions. Older or less directly relevant citations were replaced.

**Comment 10. *“‘Serum thiol-disulfide homeostasis parameters were evaluated using the methods described by Erel and Neselioglu.[8]n this method,’ Please correct typos and edit the manuscript.”***

**Response 10:** All typographical errors, including this one, have been corrected. The revised sentence now reads: **“Serum thiol–disulfide homeostasis parameters were evaluated using the method described by Erel and Neselioglu [8].”**

**Comment 11. *“Please refrain from phrases such as our and we in scientific prose.”***

**Response** **11:** The manuscript has been carefully revised to remove first-person expressions (“we,” “our”) and ensure adherence to scientific prose style. Sentences have been reformulated in the passive voice where appropriate.

We once again sincerely thank Reviewer 2 for the valuable and insightful feedback provided. The constructive suggestions not only guided us in refining the clarity, precision, and methodological rigor of our manuscript but also strengthened the overall scientific value of the work. By incorporating these comments, we believe the manuscript has been substantially improved in terms of both readability and impact. We are particularly grateful for the reviewer’s emphasis on methodological clarity and literature updates, which allowed us to better position our study within the current body of knowledge. We greatly appreciate the reviewer’s expertise and the time invested in providing such thoughtful critiques, which have meaningfully contributed to enhancing the quality and potential contribution of our research to the field.

**Reviewer 3:**

We would like to extend our sincere gratitude to Reviewer 3 for the thoughtful and encouraging evaluation of our manuscript. Your positive feedback, together with the constructive remarks, has greatly motivated us and confirmed the scientific value of our work. We deeply appreciate the recognition of our efforts to improve clarity, methodological rigor, and overall presentation. Your supportive comments not only strengthened the current version of the study but also provided us with valuable guidance for our future research.

**We would like to sincerely thank the Academic Editor and all reviewers for their time, effort, and constructive feedback during the evaluation of our manuscript. The insightful comments and valuable suggestions have been instrumental in improving the clarity, rigor, and overall quality of our work. We greatly appreciate the opportunity to revise our study in accordance with these recommendations, and we believe the manuscript has been significantly strengthened as a result. We are grateful for the careful consideration given to our submission and for the important role the editorial and review process has played in shaping this research into a more impactful scientific contribution.**

**Sincerely,**

**Assis Prof Dr. Veli Fahri PEHLİVAN**

Harran University, Faculty of Medicine, Department of Anesthesia and Reanimation Osmanbey Campus, PC 63300, Sanlıurfa, TÜRKİYE

mail:  [vfpehlivan@harran.edu.tr](mailto:%20vfpehlivan@harran.edu.tr); vfpehlivan@gmail.com
